# Aneris: A Mechanised Logic for Modular Reasoning about Distributed Systems

**DOI:** 10.1007/978-3-030-44914-8_13

**Published:** 2020-04-18

**Authors:** Morten Krogh-Jespersen, Amin Timany, Marit Edna Ohlenbusch, Simon Oddershede Gregersen, Lars Birkedal

**Affiliations:** ETH Zurich, Zurich, Switzerland; Aarhus University, Aarhus, Denmark

**Keywords:** Distributed systems, Separation logic, Higher-order logic, Concurrency, Formal verification

## Abstract

Building network-connected programs and distributed systems is a powerful way to provide scalability and availability in a digital, always-connected era. However, with great power comes great complexity. Reasoning about distributed systems is well-known to be difficult. In this paper we present Aneris, a novel framework based on separation logic supporting modular, node-local reasoning about concurrent and distributed systems. The logic is higher-order, concurrent, with higher-order store and network sockets, and is fully mechanized in the Coq proof assistant. We use our framework to verify an implementation of a load balancer that uses multi-threading to distribute load amongst multiple servers and an implementation of the *two-phase-commit* protocol with a replicated logging service as a client. The two examples certify that Aneris is well-suited for both horizontal and vertical modular reasoning.

## 1 Introduction

Reasoning about distributed systems is notoriously difficult due to their sheer complexity. This is largely the reason why previous work has traditionally focused on verification of protocols of core network components. In particular, in the context of model checking, where safety and liveness assertions [29] are considered, tools such as SPIN [9], TLA+ [23], and Mace [17] have been developed. More recently, significant contributions have been made in the field of formal proofs of *implementations* of challenging protocols, such as two-phase-commit, lease-based key-value stores, Paxos, and Raft [7, 25, 30, 35, 40]. All of these developments define domain specific languages (DSLs) specialized for distributed systems verification. Protocols and modules proven correct can be compiled to an executable, often relying on some trusted code-base.

Formal reasoning about distributed systems has often been carried out by giving an abstract model in the form of a *state transition system* or *flow-chart* in the tradition of Floyd [5], Lamport [21, 22]. A state is normally taken to be a

* This research was carried out while Amin Timany was at KU Leuven, working as a postdoctoral fellow of the Flemish research fund (FWO).

view of the global state and events are observable changes to this state. State transition systems are quite versatile and have been used in other verification applications. However, reasoning based on state transition systems often suffer from a lack of modularity due to their very global. As a consequence, separate nodes or components cannot be verified in isolation and the system has to be verified as a whole.

IronFleet [7] is the first system that supports node-local reasoning for verifying the implementation of programs that run on different nodes. In IronFleet, a distributed system is modeled by a transition system. This transition system is shown to be refined by the composition of a number of transition systems, each pertaining to one of the nodes in the system. Each node in the distributed system is shown to be correct and a refinement of its corresponding transition system. Nevertheless, IronFleet does not allow you to reason compositionally; a correctness proof for a distributed system cannot be used to show the correctness of a larger system.

Higher-order concurrent separation logics (CSLs) [3, 4, 13, 15, 18, 26, 27, 28, 33, 34, 36, 39] simplify reasoning about higher-order imperative concurrent programs by offering facilities for specifying and proving correctness of programs in a modular way. Indeed, their support for modular reasoning (a.k.a. compositional reasoning) is the key reason for their success. Disel [35] is a separation logic that does support compositional reasoning about distributed systems, allowing correctness proofs of distributed systems to be used for verifying larger systems. However, Disel struggles with node-local reasoning in that it cannot hide node-local usage of mutable state. That is, the use of internal state in nodes must be exposed in the high-level protocol of the system and changes to the internal state are only possible upon sending and receiving messages over the network.

Finally, both Disel and IronFleet restrict nodes to run only sequential programs and no node-level concurrency is supported.

In this paper we present Aneris, a framework for implementing and reasoning about functional correctness of distributed systems. Aneris is based on concurrent separation logic and supports modular reasoning with respect to both nodes (node-local reasoning) and threads within nodes (thread-local reasoning). The Aneris framework consists of a programming language, AnerisLang, for writing realistic, real-world distributed systems and a higher-order concurrent separation logic for reasoning about these systems. AnerisLang is a concurrent ML-like programming language with higher-order functions, local state, threads, and network primitives. The operational semantics of the language, naturally, involves multiple hosts (each with their own heap and multiple threads) running in a network. The Aneris logic is build on top of the Iris framework [13, 15, 18] and supports machine-verified formal proofs in the Coq proof assistant about distributed systems written in AnerisLang.

*Networking.* There are several ways of adding network primitives to a programming language. One approach is *message-passing* using first-class communication channels á la the $\pi$-calculus or using an implementation of the actor model as done in high-level languages like Erlang, Elixir, Go, and Scala. However, any such implementation is an abstraction built on top of network sockets where all data has to be serialized, data packets may be dropped, and packet reception may not follow the transmission order. Network sockets are a quintessential part of building efficient, real-world distributed systems and all major operating systems provide an application programming interface (API) to them. Likewise, AnerisLang provides support for datagram-like sockets by directly exposing a simple API with the core methods necessary for socket-based communication using the User Datagram Protocol (UDP) with duplicate protection. This allows for a wide range of real-world systems and protocols to be implemented (and verified) using the Aneris framework.

*Modular Reasoning in Aneris.* In general, there are two different ways to support modular reasoning about distributed systems corresponding to how components can be composed. Aneris enables simultaneously both:

– *Vertical composition*: when reasoning about programs within each node, one is able to compose proofs of different components to prove correctness of the whole program. For instance, the specification of a verified data structure, e.g. a concurrent queue, should suffice for verifying programs written against that data structure, independently of its implementation.
– *Horizontal composition*: at each node, a verified thread is composable with other verified threads. Similarly, a verified node is composable with other verified nodes which potentially engage in different protocols. This naturally aids implementing and verifying large-scale distributed systems.

Node-local variants of the standard rules of CSLs like, for example, the bind rule and the frame rule (as explained in Sect. 2) enable vertical reasoning. Sect. 6 showcases vertical reasoning in Aneris using a replicated distributed logging service that is implemented and verified using a separate implementation and specification of the two-phase commit protocol.

Horizontal reasoning in Aneris is achieved through the Thread-par-rule and the Node-par-rule (further explained in Sect. 2) which intuitively says that to verify a distributed system, it suffices to verify each thread and each node in isolation. This is analogous to how CSLs allow us to reason about multi-threaded programs by considering individual threads in isolation; in Aneris we extend this methodology to include both threads and nodes. Where most variants of concurrent separation logic use some form of an invariant mechanism to reason about shared-memory concurrency, we abstract the communication between nodes over the network through *socket protocols* that restrict what can be sent and received on a socket and allow us to share ownership of logical resources among nodes. Sect. 5 showcases horizontal reasoning in Aneris using an implementation and a correctness proof for a simple addition service that uses a load balancer to distribute the workload among several addition servers. Each node is verified in isolation and composed to form the final distributed system.

*Contributions.* In summary, we make the following contributions:

– We present AnerisLang, a formalized higher-order functional programming language for writing distributed systems. The language features higher-order store, node-local concurrency, and network sockets, allowing for dynamic creation and binding of sockets to addresses with serialization and deserialization primitives for encoding and parsing messages.
– We define the Aneris logic, the first higher-order concurrent separation logic with support for network sockets and with support for both node-local and thread-local reasoning.
– We introduce a simple and novel approach to specifying network protocols; a mechanism that supports separation-logic-style modular specifications of distributed systems.
– We conduct two case studies that showcase how our framework aids the implementation and verification of real-world distributed systems using compositional reasoning:
  • A replicated logging service that is implemented and verified using a separate implementation and specification of the two-phase commit protocol, demonstrating vertical compositional reasoning.
  • A load balancer that distributes work on multiple servers by means of node-local multi-threading. We use this to verify a simple addition service that uses the load balancer to distribute its requests over multiple servers, demonstrating horizontal compositional reasoning.
– We have formalized all of the theory and examples on top of Iris in the Coq proof assistant using the MoSeL framework [19]. The Coq formalization can be found online at https://iris-project.org/artifacts/2020-esop-aneris.tar.gz.

*Outline.* We start by describing the core concepts of the Aneris framework in Sec. 2. We then describe the AnerisLang programming language (Sec. 3) before presenting the Aneris logic proof rules and stating our adequacy theorem, *i.e.*, soundness of Aneris, in Sec. 4. Subsequently, we use the logic to verify a load balancer (Sec. 5) and a two-phase-commit implementation with a replicated logging client (Sec. 6). We discuss related work in Sec. 7 and conclude in Sec. 8.

## 2 The Core Concepts of Aneris

In this section we present our methodology to modular verification of distributed systems. We begin by recalling the ideas of thread-local reasoning and protocols from concurrent separation logic and explain how we lift those ideas to *node-local* reasoning. Finally, we illustrate the Aneris methodology for specifying, implementing, and verifying distributed systems by developing a simple addition service and a lock server. The distributed systems are composed of individually verified concurrently running nodes communicating asynchronously by exchanging messages that can be reordered or dropped.

### 2.1 Local and Thread-Local Reasoning

The most important feature of (concurrent) separation logic is, arguably, how it enables scalable modular reasoning about pointer-manipulating programs.

Separation logic is a resource logic, in the sense that propositions denote not only facts about the state, but *ownership* of resources. Originally, separation logic [32] was introduced for modular reasoning about the heap—i.e. the notion of resource was fixed to be logical pieces of the heap. The essential idea is that we can give a local specification $\{P\}\, e\, \{v.Q\}$ to a program $e$ involving only the *footprint* of $e$. Hence, while verifying $e$, we need not consider the possibility that another piece of code in the program might interfere with $e$; the program $e$ can be verified without concern for the environment in which $e$ may occur. Local specifications can then be lifted to more global specifications by framing and binding:

$$\frac{\{P\}\, e\, \{v.Q\}}{\{P * R\}\, e\, \{v.Q * R\}} \qquad\qquad \frac{\{P\}\, e\, \{v.Q\} \qquad \forall v. \{Q\}\, K[v]\, \{w.R\}}{\{P\}\, K[e]\, \{w.R\}}$$

where $K$ denotes an evaluation context. The symbol $*$ denotes separating conjunction. Intuitively, $P * Q$ holds for a given resource (in this case a heap) if it can be divided into two disjoint resources such that $P$ holds for one and $Q$ holds for the other. Thus, the frame rule essentially says that executing $e$ for which we know $\{P\}\, e\, \{x.Q\}$ cannot possibly affect parts of the heap that are *separate* from its footprint. Another related separation logic connective is $-\!\!*$, the separating implication. Proposition $P -\!\!* Q$ describes a resource that, combined with a disjoint resource satisfying $P$, results in a resource satisfying $Q$.

Since its introduction, separation logic has been extended to resources beyond heaps and with more sophisticated mechanisms for modular control of interference. Concurrent separation logics (CSLs) [28] allow reasoning about concurrent programs and a preeminent feature of these program logics is again the support for modular reasoning, in this case with respect to concurrency through *thread-local* reasoning. When reasoning about a concurrent program we consider threads one at a time and need not reason about interleavings of threads explicitly. In a way, our frame here includes, in addition to the shared fragments of the heap and other resources, the execution of other threads which can be interleaved throughout the execution of the thread being verified. This can be seen from the following disjoint concurrency rule:

$$\textsc{Thread-par}\quad \frac{\{P_1\}\, \langle n; e_1\rangle\, \{v.Q_1\} \qquad \{P_2\}\, \langle n; e_2\rangle\, \{v.Q_2\}}{\{P_1 * P_2\}\, \langle n; e_1 \mid\mid e_2\rangle\, \{v.\exists v_1, v_2. v = (v_1, v_2) * Q_1[v_1/v] * Q_2[v_2/v]\}}$$

where $e_1 \mid\mid e_2$ denotes parallel composition of expressions $e_1$ and $e_2$ and we use the notation $\langle n; e\rangle$ to denote an expression $e$ running on a node with identifier $n$.[1]

Inevitably, at some point threads typically have to communicate with one another through some kind of shared state, an unavoidable form of interference. The original CSL used a simple form of resource invariant in which ownership of a shared resource can be transferred between threads.

[1] In a language with fork-based concurrency, the parallel composition operator is an easily defined construct and the rule is derivable from a more general fork-rule.

A notable program logic in the family of concurrent separation logics is Iris that is specifically designed for reasoning about programs written in concurrent higher-order imperative programming languages. Iris has already proven to be versatile for reasoning about a number of sophisticated properties of programming languages [12, 16, 37]. In order to support modular reasoning about concurrent programs Iris features (1) *impredicative invariants* for expressing protocols on shared state among multiple threads and (2) allows for encoding of *higher-order ghost state* using a form of partial commutative monoids for reasoning about resources. We will give examples of these features and explain them in more detail as needed.

## 2.2 Node-Local Reasoning

Programs written in AnerisLang are higher-order imperative concurrent programs that run on multiple nodes in a distributed system. When reasoning about distributed systems in Aneris, alongside heap-local and thread-local reasoning, we also reason *node-locally*. When proving correctness of AnerisLang programs we reason about each node of the system in isolation, akin to how we in CSLs reason about each thread in isolation.

By virtue of building on Iris, reasoning in Aneris is naturally modular with respect to separation logic frames and with respect to threads. What Aneris adds on top of this is support for *node-local* reasoning about programs. This is expressed by the following rule:

Node-par

$$\frac{\{P_1 * \mathsf{IsNode}(n_1) * \mathsf{FreePorts}(ip_1, \mathfrak{P})\}\ \langle n_1; e_1\rangle\ \{\mathsf{True}\} \qquad \{P_2 * \mathsf{IsNode}(n_2) * \mathsf{FreePorts}(ip_2, \mathfrak{P})\}\ \langle n_2; e_2\rangle\ \{\mathsf{True}\}}{\{P_1 * P_2 * \mathsf{FreeIp}(ip_1) * \mathsf{FreeIp}(ip_2)\}\ \langle \mathfrak{S}; (n_1; ip_1; e_1) \mid\mid\mid (n_2; ip_2; e_2)\rangle\ \{\mathsf{True}\}}$$

where $|||$ denotes parallel composition of two nodes with identifier $n_1$ and $n_2$ running expressions $e_1$ and $e_2$ with IP addresses $ip_1$ and $ip_2$.[2] The set $\mathfrak{P} = \{p \mid 0 \leq p \leq 65535\}$ denotes a finite set of ports.

Note that only a distinguished system node $\mathfrak{S}$ can start new nodes (as elaborated on in Sect. 3). In Aneris, the execution of the distributed system starts with the execution of $\mathfrak{S}$ as the only node in the system. In order to start a new node associated with ip address $ip$ one provides the resource $\mathsf{FreeIp}(ip)$ which indicates that $ip$ is not used by other nodes. The node can then rely on the fact that when it starts, all ports on $ip$ are available. The resource $\mathsf{IsNode}(n)$ indicates that the node $n$ is a node in the system and keeps track of abstract state related to our modeling of node $n$'s heap and allocated sockets. To facilitate modular reasoning, free ports can be split: if $A \cap B = \emptyset$ then $\mathsf{FreePorts}(ip, A) * \mathsf{FreePorts}(ip, B) \dashv\vdash \mathsf{FreePorts}(ip, A \cup B)$ where $\dashv\vdash$ denotes

[2] In the same way as the parallel composition rule is derived from a more general fork-based rule, this composition rule is also an instance of a more general rule for spawning nodes shown in Sect. 3.

logical equivalence of Aneris propositions (of type *iProp*). We will use $\mathsf{FreePort}(a)$ as shorthand for $\mathsf{FreePorts}(ip, \{p\})$ where $a = (ip, p)$.

Finally, observe that the node-local postconditions are simply True, in contrast to the arbitrary thread-local postconditions in the Thread-par-rule that carry over to the main thread. In the concurrent setting, shared memory provides reliable communication and synchronization between the child threads and the main thread; in the rule for parallel composition, the main thread will wait for the two child processes to finish. In the distributed setting, there are no such guarantees and nodes are separate entities that cannot synchronize with the distinguished system node.

*Socket Protocols.* Similar to how classical CSLs introduce the concept of resource invariants for expressing protocols on shared state among multiple threads, we introduce the simple and novel concept of *socket protocols* for expressing protocols among multiple nodes. With each socket address—a pair of an IP address and a port—a protocol is associated, which restricts what can be communicated on that socket.

A socket protocol is a predicate $\Phi : \mathit{Message} \to \mathit{iProp}$ on incoming messages received on a particular socket. One can think of this as a form of rely-guarantee reasoning since the socket protocol will be used to restrict the distributed environment's interference with a node on a particular socket. In Aneris we write $a \Mapsto \Phi$ to mean that socket address $a$ is governed by the protocol $\Phi$. In particular, if $a \Mapsto \Phi$ and $a \Mapsto \Psi$ then $\Phi$ and $\Psi$ are equivalent.[3] Moreover, the proposition is duplicable: $a \Mapsto \Phi \dashv\vdash a \Mapsto \Phi * a \Mapsto \Phi$.

Conceptually, a socket is an abstract representation of a handle for a local endpoint of some channel. We further restrict channels to use the User Datagram Protocol (UDP) which is *asynchronous*, *connectionless*, and *stateless*. In accordance with UDP, Aneris provides no guarantee of delivery or ordering although we assume duplicate protection. We assume duplicate protection to simplify our examples, as otherwise the code of all of our examples would have to be adapted to cope with duplication of messages. One can think of sockets in Aneris as open-ended multi-party communication channels without synchronization.

It is noteworthy that inter-process communication can happen in two ways. Thread-concurrent programs can communicate both through the shared heap and by sending messages through sockets. For memory-separated programs running on different nodes all communication is by message-passing.

In the logic, we consider both *static* and *dynamic* socket addresses. This distinction is entirely abstract and at the level of the logic. Static addresses come with primordial protocols, agreed upon before starting the distributed system, whereas dynamic addresses do not. Protocols on static addresses are primarily intended for addresses pointing to nodes that offer a service.

To distinguish between static and dynamic addresses, we use a resource $\mathsf{Fixed}(A)$ which denotes that the addresses in $A$ are static and should have a fixed

[3] The predicate equivalence is under a later modality in order to avoid self-referential paradoxes. We omit it for the sake of presentation as this is an orthogonal issue.

interpretation. This proposition expresses knowledge without asserting ownership of resources and is duplicable: $\mathsf{Fixed}(A) \dashv\vdash \mathsf{Fixed}(A) * \mathsf{Fixed}(A)$.

Corresponding to the two kinds of addresses we have two different rules, Socketbind-static and Socketbind-dynamic, for binding an address to a socket as seen below. Both rules consume an instance of $\mathsf{Fixed}(A)$ and $\mathsf{FreePort}(a)$ as well as a resource $z \hookrightarrow_n \mathsf{None}$. The latter keeps track of the address associated with the socket handle $z$ on node $n$ and ensures that the socket is bound only once as further explained in Sect. 4. Notice that the protocol $\Phi$ in Socketbind-dynamic can be freely chosen.

Socketbind-static

$$\{\mathsf{Fixed}(A) * a \in A * \mathsf{FreePort}(a) * z \hookrightarrow_n \mathsf{None}\}$$

$$\langle n; \texttt{socketbind}\ z\ a\rangle$$

$$\{x.\, x = 0 * z \hookrightarrow_n \mathsf{Some}\ a\}$$

Socketbind-dynamic

$$\{\mathsf{Fixed}(A) * a \notin A * \mathsf{FreePort}(a) * z \hookrightarrow_n \mathsf{None}\}$$

$$\langle n; \texttt{socketbind}\ z\ a\rangle$$

$$\{x.\, x = 0 * z \hookrightarrow_n \mathsf{Some}\ a * a \Mapsto \Phi\}$$

In the remainder of the paper we will use the following shorthands in order to simplify the presentation of our specifications.

$$\mathsf{Static}(a, A, \Phi) \triangleq \mathsf{Fixed}(A) * a \in A * \mathsf{FreePort}(a) * a \Mapsto \Phi$$

$$\mathsf{Dynamic}(a, A) \triangleq \mathsf{Fixed}(A) * a \notin A * \mathsf{FreePort}(a)$$

### 2.3 Example: An Addition Service

To illustrate node-local reasoning, socket protocols, and the Aneris methodology for specifying, implementing, and verifying distributed systems we develop a simple addition service that offers to add numbers for clients.

Fig. 1 depicts an implementation of a server and a client written in AnerisLang. Notice that the programs look as if they were written in a realistic functional language with sockets like OCaml. Messages are strings to make programming with sockets easier (similar to `send_substring` in the Unix module in OCaml).

The server is parameterized over an address on which it will listen for requests. The server allocates a new socket and binds the address to the socket. Then the server starts listening for an incoming message on the socket, calling a handler function on the message, if any. The handler function will deserialize the message, perform the addition, serialize the result, and return it to the sender before recursively listening for new messages.

The client is parameterized over two numbers to compute on, a server address, and a client address. The client allocates a new socket, binds the address to the socket, and serializes the two numbers. In the end, it sends the serialized message

```
rec server a =
  let skt = socket () in
  socketbind skt a;
  listen skt (rec handler msg from =
    let m = deserialize msg in
    let res = serialize (π₁ m + π₂ m) in
    sendto skt res from;
    listen skt handler)
```

```
rec client x y srv a =
  let skt = socket () in
  socketbind skt a;
  let m = serialize (x, y) in
  sendto skt m srv;
  let res = listenwait skt in
  deserialize (π₁ res)
```

**Fig. 1.** An implementation of an addition service and a client written in AnerisLang. `listen` and `listenwait` are convenient helper functions to be found in the appendix [20].

to the server address using the socket and waits for a response, projecting out the result of the addition on arrival and deserializing it.

In order to give the server code a specification we will fix a primordial socket protocol that will govern the address given to the server. The protocol will spell out how the server relies on the socket. We will use $\mathsf{from}(m)$ and $\mathsf{body}(m)$ for projections of the sender and the message body, respectively, from the message $m$. We define $\Phi_{add}$ as follows:

$$\begin{aligned}\Phi_{add}(m) \triangleq \exists \Psi, x, y.\, \mathsf{from}(m) \Mapsto \Psi * \mathsf{body}(m) = \mathit{serialize}(x, y) \,*\\ \forall m', \mathsf{body}(m') = \mathit{serialize}(x + y) \mathrel{-\!\!*} \Psi(m')\end{aligned}$$

Intuitively, the protocol demands that the sender of a message $m$ is governed by some protocol $\Psi$ and that the message body $\mathsf{body}(m)$ must be the serialization of two numbers $x$ and $y$. Moreover, the sender's protocol must be satisfied if the serialization of $x + y$ is sent as a response.

Using $\Phi_{add}$ as the socket protocol, we can give `server` the specification

$$\{\mathsf{Static}(a, A, \Phi_{add}) * \mathsf{IsNode}(n)\}\ \langle n; \texttt{server}\ a\rangle\ \{\mathsf{False}\}.$$

The postcondition is allowed to be False as the program does not terminate. The triple guarantees safety which, among others, means that *if* the server responds to communication on address $a$ it does so according to $\Phi_{add}$.

Similarly, using $\Phi_{add}$ as a primordial protocol for the server address, we can also give `client` a specification

$$\begin{aligned}&\{srv \Mapsto \Phi_{add} * srv \in A * \mathsf{Dynamic}(a, A) * \mathsf{IsNode}(m)\}\\ &\quad\langle m; \texttt{client}\ x\ y\ srv\ a\rangle\\ &\{v.v = x + y\}\end{aligned}$$

that showcases how the client is able to conclude that the response from the server is the sum of the numbers it sent to it. In the proof, when binding $a$ to the socket using SOCKETBIND-DYNAMIC, we introduce the proposition $a \Mapsto \Phi_{client}$ where

$$\Phi_{client}(m) \triangleq \mathsf{body}(m) = \mathit{serialize}(x + y)$$

and use it to instantiate $\Psi$ when satisfying $\Phi_{add}$. Using the two specifications and the NODE-PAR-rule it is straightforward to specify and verify a distributed system composed of, e.g., a server and multiple clients.

### 2.4 Example: A Lock Server

Mutual exclusion in distributed systems is often a necessity and there are many different approaches for providing it. The simplest solution is a centralized algorithm with a single node acting as the coordinator. We will develop this example to showcase a more interesting protocol that relies on ownership transfer of spatial resources between nodes to ensure correctness.

The code for a centralized lock server implementation is shown in Fig. 2.

```
rec lockserver a =
  let lock = ref NONE in
  let skt = socket () in
  socketbind skt a;
  listen skt (rec handler msg from =
    if (msg = "LOCK") then
      match !lock with
        NONE => lock ← SOME (); sendto skt "YES" from
      | SOME _ => sendto skt "NO" from
      end
    else lock ← NONE; sendto skt "RELEASED" from
    listen skt handler)
```

**Fig. 2.** A lock server in AnerisLang.

The lock server declares a node-local variable `lock` to keep track of whether the lock is taken or not. It allocates a socket, binds the input address to the socket and continuously listens for incoming messages. When a `"LOCK"` message arrives and the lock is available, the lock gets taken and the server responds `"YES"`. If the lock was already taken, the server will respond `"NO"`. Finally, if the message was not `"LOCK"`, the lock is released and the server responds with `"RELEASED"`.

Our specification of the lock server will be inspired by how a lock can be specified in concurrent separation logic. Thus we first recall how such a specification usually looks like.

Conceptually, a lock can either be unlocked or locked, as described by a two-state labeled transition system.

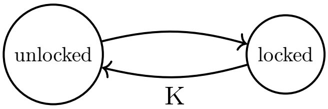

In concurrent separation logic, the lock specification does not describe this transition system directly, but instead focuses on the resources needed for the transitions to take place. In the case of the lock, the resources are simply a non-duplicable resource $K$, which is needed in order to call the lock's release method. Intuitively, this resource corresponds to the key of the lock.

A typical concurrent separation logic specification for a spin lock module looks roughly like the following:

$$\begin{aligned}
&\exists\, \text{isLock}\,. \\
\wedge\quad & \forall v, K.\ \text{isLock}(v,K) \dashv\vdash \text{isLock}(v,K) * \text{isLock}(v,K) \\
\wedge\quad & \forall v, K.\ \text{isLock}(v,K) \vdash K * K \Rightarrow \mathsf{False} \\
\wedge\quad & \{\mathsf{True}\}\ \text{newLock}\ \ ()\,\{v.\ \exists K.\ \text{isLock}(v,K)\} \\
\wedge\quad & \forall v.\,\{\text{isLock}(v,K)\}\ \text{acquire}\,v\,\{v.K\} \\
\wedge\quad & \forall v.\,\{\text{isLock}(v,K) * K\}\ \text{release}\,v\,\{\mathsf{True}\}
\end{aligned}$$

The intuitive reading of such a specification is:

- Calling newLock will lead to the duplicable knowledge of the return value $v$ being a lock.
- Knowing that a value is a lock, a thread can try to acquire the lock and when it eventually succeeds it will get the key $K$.
- Only a thread holding this key is allowed to call release.

Sharing of the lock among several threads is achieved by the isLock predicate being duplicable. Mutual exclusion is ensured by the last bullet point together with the requirement of $K$ being non-duplicable whenever we have isLock$(v, K)$. For a leisurely introduction to such specifications, the reader may consult Birkedal and Bizjak [1].

Let us now return to the distributed lock synchronization. To give clients the possibility of interacting with the lock server as they would with such a concurrent lock module, the specification for the lock server will look like follows.

$$\{K * \mathsf{Static}(a, A, \Phi_{lock})\}\ \langle n; \mathtt{lockserver}\ a\rangle\ \{\mathsf{False}\}.$$

This specification simply states that a lock server should have a primordial protocol $\Phi_{lock}$ and that it needs the key resource to begin with. To allow for the desired interaction with the server, we define the socket protocol $\Phi_{lock}$ as follows:

$$\begin{aligned}
acq(m, \Psi) &\triangleq (\mathsf{body}(m) = \text{"}\mathtt{LOCK}\text{"}) * \\
&\qquad \forall m'.\,(\mathsf{body}(m') = \text{"}\mathtt{NO}\text{"}) \vee (\mathsf{body}(m') = \text{"}\mathtt{YES}\text{"} * K) \twoheadrightarrow \Psi(m') \\
rel(m, \Psi) &\triangleq (\mathsf{body}(m) = \text{"}\mathtt{RELEASE}\text{"}) * K * \\
&\qquad \forall m'.\,(\mathsf{body}(m') = \text{"}\mathtt{RELEASED}\text{"}) \twoheadrightarrow \Psi(m') \\
\Phi_{lock}(m) &\triangleq \exists \Psi.\,\mathsf{from}(m) \Mapsto \Psi * (acq(m, \Psi) \vee rel(m, \Psi))
\end{aligned}$$

The protocol $\Phi_{lock}$ demands that a client of the lock has to be bound to some protocol $\Psi$ and that the server can receive two types of messages fulfilling either $acq(m, \Psi)$ or $rel(m, \Psi)$. These correspond to the module's two methods acquire and release respectively. In the case of a `"LOCK"` message, the server will answer either `"NO"` or `"YES"` along with the key resource. In either case, the answer should suffice for fulfilling the client protocol $\Psi$.

Receiving a "RELEASE" request is similar, but the important part is that we require a client to send the key resource $K$ along with the message, which ensures that only the current holder can release the lock.

One difference between the distributed and the concurrent specification is that we allow for the distributed lock to directly deny access. The client can use a simple loop, asking for the lock until it is acquired, if it wishes to wait until the lock can be acquired.

There are several interesting observations one can make about the lock server example: (1) The lock server can allocate, read, and write node-local references but these are hidden in the specification. (2) There are no channel descriptors or assertions on the socket in the code. (3) The lock server provides mutual exclusion by requiring clients to satisfy a sufficient protocol.

## 3 AnerisLang

AnerisLang is an untyped functional language with higher-order functions, fork-based concurrency, higher-order mutable references, and primitives for communicating over network sockets. The syntax is as follows:

$$
\begin{aligned}
v \in \mathit{Val} &::= () \mid b \mid i \mid s \mid \ell \mid z \mid \mathtt{rec}\, f\, x = e \mid \ldots \\
e \in \mathit{Expr} &::= v \mid x \mid \mathtt{rec}\, f\, x = e \mid e_1\ e_2 \mid \mathtt{ref}\, e \mid\ !\, e \mid e_1 \leftarrow e_2 \mid \mathtt{cas}\, e_1\, e_2\, e_3 \\
&\mid \mathtt{find}\ e_1\ e_2\ e_3 \mid \mathtt{substring}\ e_1\ e_2\ e_3 \mid \mathtt{i2s}\ e \mid \mathtt{s2i}\ e \\
&\mid \mathtt{fork}\ \{e\} \mid \mathtt{start}\ \{n; ip; e\} \mid \mathtt{makeaddress}\ e_1\ e_2 \\
&\mid \mathtt{socket}\ e \mid \mathtt{socketbind}\ e_1\ e_2 \mid \mathtt{sendto}\ e_1\ e_2\ e_3 \mid \mathtt{receivefrom}\ e \mid \ldots
\end{aligned}
$$

We omit the usual operations on pairs, sums, booleans $b \in \mathbb{B}$, and integers $i \in \mathbb{Z}$ which are all standard. We introduce the following syntactic sugar: lambda abstractions $\lambda x.\, e$ defined as $\mathtt{rec}\ \_\ x = e$, let-bindings $\mathtt{let}\, x = e_1\, \mathtt{in}\, e_2$ defined as $(\lambda x.\, e_2)(e_1)$, and sequencing $e_1; e_2$ defined as $\mathtt{let}\ \_ = e_1\, \mathtt{in}\, e_2$.

We have the usual operations on locations $\ell \in \mathit{Loc}$ in the heap: $\mathtt{ref}\, v$ for allocating a new reference, $!\,\ell$ for dereferencing, and $\ell \leftarrow v$ for assignment. $\mathtt{cas}\, \ell\, v_1\, v_2$ is an atomic compare-and-set operation used to achieve synchronization between threads on a specific memory location $\ell$. Operationally, it tests whether $\ell$ has value $v_1$ and if so, updates the location to $v_2$, returning a boolean indicating whether the swap succeeded or not.

The operation find finds the index of a particular substring in a string $s \in \mathit{String}$ and substring splits a string at given indices, producing the corresponding substring. i2s and s2i convert between integers and strings. These operations are mainly used for serialization and deserialization purposes.

The expression $\mathtt{fork}\ \{e\}$ forks off a new (node-local) thread and $\mathtt{start}\ \{n; ip; e\}$ will spawn a new node $n \in \mathit{Node}$ with ip address $ip \in \mathit{Ip}$ running the program $e$. Note that it is only at the bootstrapping phase of a distributed system that a special system-node $\mathfrak{S}$ will be able to spawn nodes.

We use $z \in \mathit{Handle}$ to range over socket handles created by the socket operation. makeaddress constructs an address given an ip address and a port, and the network primitives `socketbind`, `sendto`, and `receivefrom` correspond to the similar BSD-socket API methods.

*Operational Semantics.* We define the operational semantics of AnerisLang in three stages.

We first define a node-local, thread-local, head step reduction $(e, h) \rightsquigarrow (e', h')$ for $e, e' \in \mathit{Expr}$ and $h, h' \in \mathit{Loc} \xrightarrow{\text{fin}} \mathit{Val}$ that handles all pure and heap-related node-local reductions. All rules of the relation are standard.

Next, the node-local head step reduction induces a network-aware head step reduction $(\langle n; e \rangle, \Sigma) \rightarrow (\langle n; e' \rangle, \Sigma')$.

$$\frac{(e, h) \rightsquigarrow (e', h')}{\langle n; e \rangle, (\mathcal{H}[n \mapsto h], \mathcal{S}, \mathcal{P}, \mathcal{M}) \rightarrow \langle n; e' \rangle, (\mathcal{H}[n \mapsto h'], \mathcal{S}, \mathcal{P}, \mathcal{M})}.$$

Here $n \in \mathit{Node}$ denotes a node identifier and $\Sigma, \Sigma' \in \mathit{NetworkState}$ the global network state. Elements of $\mathit{NetworkState}$ are tuples $(\mathcal{H}, \mathcal{S}, \mathcal{P}, \mathcal{M})$ tracking heaps $\mathcal{H} \in \mathit{Node} \xrightarrow{\text{fin}} \mathit{Heap}$ and sockets $\mathcal{S} \in \mathit{Node} \xrightarrow{\text{fin}} \mathit{Handle} \xrightarrow{\text{fin}} \mathit{Option\ Address}$ for all nodes, ports in use $\mathcal{P} \in \mathit{Ip} \xrightarrow{\text{fin}} \wp^{\text{fin}}(\mathit{Port})$, and messages sent $\mathcal{M} \in \mathit{Id} \xrightarrow{\text{fin}} \mathit{Message}$. The induced network-aware reduction is furthermore extended with rules for the network primitives as seen in Fig. 3. The `socket` operation allocates a new

$$\frac{z \notin \mathrm{dom}(\mathcal{S}(n)) \qquad \mathcal{S}' = \mathcal{S}[n \mapsto \mathcal{S}(n)[z \mapsto \mathsf{None}]]}{\langle n; \mathtt{socket}\ () \rangle, (\mathcal{H}, \mathcal{S}, \mathcal{P}, \mathcal{M}) \rightarrow \langle n; z \rangle, (\mathcal{H}, \mathcal{S}', \mathcal{P}, \mathcal{M})}$$

$$\frac{\begin{array}{c}\mathcal{S}(n)(z) = \mathsf{None} \\ p \notin \mathcal{P}(ip) \qquad \mathcal{S}' = \mathcal{S}[n \mapsto \mathcal{S}(n)[z \mapsto \mathsf{Some}\ (ip, p)]] \qquad \mathcal{P}' = \mathcal{P}[ip \mapsto \mathcal{P}(ip) \cup \{p\}]\end{array}}{\langle n; \mathtt{socketbind}\ z\ (ip, p) \rangle, (\mathcal{H}, \mathcal{S}, \mathcal{P}, \mathcal{M}) \rightarrow \langle n; 0 \rangle, (\mathcal{H}, \mathcal{S}', \mathcal{P}', \mathcal{M})}$$

$$\frac{\mathcal{S}(n)(z) = \mathsf{Some}\ \mathit{from} \qquad i \notin \mathit{dom}(\mathcal{M}) \qquad \mathcal{M}' = \mathcal{M}[i \mapsto (\mathit{from}, \mathit{to}, \mathit{msg}, \textsc{Sent})]}{\langle n; \mathtt{sendto}\ z\ \mathit{msg}\ \mathit{to} \rangle, (\mathcal{H}, \mathcal{S}, \mathcal{P}, \mathcal{M}) \rightarrow \langle n; |\mathit{msg}| \rangle, (\mathcal{H}, \mathcal{S}, \mathcal{P}, \mathcal{M}')}$$

$$\frac{\begin{array}{c}\mathcal{S}(n)(z) = \mathsf{Some}\ \mathit{to} \\ \mathcal{M}(i) = (\mathit{from}, \mathit{to}, \mathit{msg}, \textsc{Sent}) \qquad \mathcal{M}' = \mathcal{M}[i \mapsto (\mathit{from}, \mathit{to}, \mathit{msg}, \textsc{Received})]\end{array}}{\langle n; \mathtt{receivefrom}\ z \rangle, (\mathcal{H}, \mathcal{S}, \mathcal{P}, \mathcal{M}) \rightarrow \langle n; \mathtt{Some}\ (\mathit{msg}, \mathit{from}) \rangle, (\mathcal{H}, \mathcal{S}, \mathcal{P}, \mathcal{M}')}$$

$$\frac{\mathcal{S}(n)(z) = \mathsf{Some}\ \mathit{to}}{\langle n; \mathtt{receivefrom}\ z \rangle, (\mathcal{H}, \mathcal{S}, \mathcal{P}, \mathcal{M}) \rightarrow \langle n; \mathtt{None} \rangle, (\mathcal{H}, \mathcal{S}, \mathcal{P}, \mathcal{M})}$$

**Fig. 3.** An excerpt of the rules for network-aware head reduction.

unbound socket using a fresh handle $z$ for a node $n$ and `socketbind` binds a socket address $a$ to an unbound socket $z$ if the address and port $p$ is not already in use. Hereafter, the port is no longer available in $\mathcal{P}'(ip)$. For bound sockets, `sendto` sends a message $\mathit{msg}$ to a destination address $\mathit{to}$ from the sender's address

*from* found in the bound socket. The message is assigned a unique identifier and tagged with a status flag Sent indicating that the message has been sent and not received. The operation returns the number of characters sent.

To model possibly dropped or delayed messages we introduce two rules for receiving messages using the `receivefrom` operation that on a bound socket either returns a previously unreceived message or nothing. If a message is received the status flag of the message is updated to Received

Third and finally, using standard *call-by-value right-to-left evaluation contexts* $K \in Ectx$ we lift the node-local head reduction to a *distributed systems* reduction $\twoheadrightarrow$ shown below. We write $\twoheadrightarrow^*$ for its reflexive-transitive closure. The distributed systems relation reduces by picking a thread on any node or forking off a new thread on a node.

$$\frac{(\langle n; e\rangle, \Sigma) \to (\langle n; e'\rangle, \Sigma')}{(\boldsymbol{T}_1 \mathbin{+\!\!+} [\langle n; K[e]\rangle] \mathbin{+\!\!+} \boldsymbol{T}_2, \Sigma) \twoheadrightarrow (\boldsymbol{T}_1 \mathbin{+\!\!+} [\langle n; K[e']\rangle] \mathbin{+\!\!+} \boldsymbol{T}_2; \Sigma')}$$

$$(\boldsymbol{T}_1 \mathbin{+\!\!+} [\langle n; K[\texttt{fork}\ \{e\}]\rangle] \mathbin{+\!\!+} \boldsymbol{T}_2, \Sigma) \twoheadrightarrow (\boldsymbol{T}_1 \mathbin{+\!\!+} [\langle n; K[()]\rangle] \mathbin{+\!\!+} \boldsymbol{T}_2 \mathbin{+\!\!+} [\langle n; e\rangle], \Sigma)$$

## 4 The Aneris Logic

As a consequence of building on the Iris framework, the Aneris logic features all the usual connectives and rules of higher-order separation logic, some of which are shown in the grammar below.[4] The full expressiveness of the logic can be exploited when giving specifications to programs or stating protocols.

$$\begin{aligned} P, Q \in iProp ::= {} & \mathsf{True} \mid \mathsf{False} \mid P \wedge Q \mid P \vee Q \mid P \Rightarrow Q \mid \\ & \forall x.\, P \mid \exists x.\, P \mid P * Q \mid P \mathrel{-\!\!*} Q \mid t = u \mid \\ & \ell \mapsto_n v \mid \boxed{P} \mid \left[\bar{a}\right]^{\gamma} \mid \{P\}\,\langle n; e\rangle\,\{x.\ Q\} \mid \ \ldots \end{aligned}$$

Note that in Aneris the usual points-to connective about the heap, $\ell \mapsto_n v$, is indexed by a node identifier $n \in Node$, asserting ownership of the singleton heap mapping $\ell$ to $v$ on node $n$.

The logic features (impredicative) invariants $\boxed{P}$ and user-definable ghost state via the proposition $\left[\bar{a}\right]^{\gamma}$, which asserts ownership of a piece of ghost state $a$ at ghost location $\gamma$. The logical support for user-defined invariants and ghost state allows one to relate (ghost and physical) resources to each other; this is vital for our specifications as will become evident in Sect. 5 and Sect. 6. We refer to Jung et al. [14] for a more thorough treatment of user-defined ghost state.

To reason about AnerisLang programs, the logic features Hoare triples.[5] The intuitive reading of the Hoare triple $\{P\}\,\langle n; e\rangle\,\{x.\ Q\}$ is that if the program $e$ on

[4] To avoid the issue of reentrancy, invariants are annotated with a *namespace* and Hoare triples with a *mask*. We omit both for the sake of presentation as they are orthogonal issues.

[5] In both Iris and Aneris the notion of a Hoare triple is defined in terms of a *weakest precondition* but this will not be important for the remainder of this paper.

node $n$ is run in a distributed system $s$ satisfying $P$, then the computation does not get stuck and, moreover, if it terminates with a value $v$ and in a system $s'$, then $s'$ satisfies $Q[v/x]$. In other words, a Hoare triple implies safety and states that all spatial resources that are used by $e$ are contained in the precondition $P$.

In contrast to spatial propositions that express *ownership*, e.g., $\ell \mapsto_n v$, propositions like $\boxed{P}$ and $\{P\}\,\langle n; e\rangle\,\{x.\, Q\}$ express *knowledge* of properties that, once true, hold true forever. We call this class of propositions *persistent*. Persistent propositions $P$ can be freely duplicated: $P \dashv\vdash P * P$.

## 4.1 The Program Logic

The Aneris proof rules include the usual rules of concurrent separation logic for Hoare triples, allowing formal reasoning about node-local pure computations, manipulations of the the heap, and forking of threads. Expressions $e$ are annotated with a node identifier $n$, but the rules are otherwise standard.

To reason about individual nodes in a distributed system in isolation, Aneris introduces the following rule:

Start

$$\frac{\{P * \mathsf{IsNode}(n) * \mathsf{FreePorts}(ip, \mathfrak{P})\}\,\langle n; e\rangle\,\{\mathsf{True}\}}{\{P * \mathsf{FreeIp}(ip)\}\,\langle \mathfrak{S}; \mathtt{start}\ \{n; ip; e\}\rangle\,\{x.\, x = ()\}}$$

where $\mathfrak{P} = \{p \mid 0 \leq p \leq 65535\}$. This rule is the key rule allowing node-local reasoning; the rule expresses exactly that to reason about a distributed system it suffices to reason about each node in isolation.

As described in Sect. 3, only the distinguished system node $\mathfrak{S}$ can start new nodes—this is also reflected in the Start-rule. In order to start a new node associated with IP address $ip$, the resource $\mathsf{FreeIp}(ip)$ is provided. This indicates that $ip$ is not used by other nodes. When reasoning about the node $n$, the proof can rely on all ports on $ip$ being available. The resource $\mathsf{IsNode}(n)$ indicates that the node $n$ is a valid node in the system and keeps track of abstract state related to the modeling of node $n$'s heap and sockets. $\mathsf{IsNode}(n)$ is persistent and hence duplicable.

*Network Communication.* To reason about network communication in a distributed system, the logic includes a series of rules for reasoning about socket manipulation: allocation of sockets, binding of addresses to sockets, sending via sockets, and receiving from sockets.

To allocate a socket it suffices to prove that the node $n$ is valid by providing the $\mathsf{IsNode}(n)$ resource. In return, an unbound socket resource $z \hookrightarrow_n \mathsf{None}$ is given.

Socket

$$\{\mathsf{IsNode}(n)\}\,\langle n; \mathtt{socket}\ ()\rangle\,\{z.\, z \hookrightarrow_n \mathsf{None}\}$$

The socket resource $z \hookrightarrow_n o$ keeps track of the address associated with the socket handle $z$ on node $n$ and takes part in ensuring that the socket is bound only once. It behaves similarly to the points-to connective for the heap, e.g., $z \hookrightarrow_n o * z \hookrightarrow_n o' \Rightarrow \mathsf{False}$.

As briefly touched upon in Sect. 2, the logic offers two different rules for binding an address to a socket depending on whether or not the address has a (at the level of the logic) primordial, agreed upon protocol. To distinguish between such static and dynamic addresses, we use a persistent resource $\mathsf{Fixed}(A)$ to keep track of the set of addresses that have a fixed socket protocol.

To reason about a static address binding to a socket $z$ it suffices to show that the address $a$ being bound has a fixed interpretation (by being in the "fixed" set), that the port of the address is free, and that the socket is not bound.

Socketbind-static

$$\{\mathsf{Fixed}(A) * a \in A * \mathsf{FreePort}(a) * z \hookrightarrow_n \mathsf{None}\}$$
$$\langle n; \texttt{socketbind}\ z\ a\rangle$$
$$\{x.\, x = 0 * z \hookrightarrow_n \mathsf{Some}\ a\}$$

In accordance with the BSD-socket API, the bind operation returns the integer 0 and the socket resource gets updated, reflecting the fact that the binding took place.

The rule for dynamic address binding is similar but the address $a$ should not have a fixed interpretation. Moreover, the user of the logic is free to pick the socket protocol $\Phi$ to govern address $a$.

Socketbind-dynamic

$$\{\mathsf{Fixed}(A) * a \notin A * \mathsf{FreePort}(a) * z \hookrightarrow_n \mathsf{None}\}$$
$$\langle n; \texttt{socketbind}\ z\ a\rangle$$
$$\{x.\ x = 0 * z \hookrightarrow_n \mathsf{Some}\ a * a \Mapsto \Phi\}$$

To reason about sending a message on a socket $z$ it suffices to show that $z$ is bound, that the destination of the message is governed by a protocol $\Phi$, and that the message satisfies the protocol.

Sendto

$$\{z \hookrightarrow_n \mathsf{Some}\ \mathit{from} * \mathit{to} \Mapsto \Phi * \Phi((\mathit{from}, \mathit{to}, \mathit{msg}, \textsc{Sent}))\}$$
$$\langle n; \texttt{sendto}\ z\ \mathit{msg}\ \mathit{to}\rangle$$
$$\{x.\ x = |\mathit{msg}| * z \hookrightarrow_n \mathsf{Some}\ \mathit{from}\}$$

Finally, to reason about receiving a message on a socket $z$ the socket must be bound to an address governed by a protocol $\Phi$.

Receivefrom

$$\{z \hookrightarrow_n \mathsf{Some}\ \mathit{to} * \mathit{to} \Mapsto \Phi\}$$
$$\langle n; \texttt{receivefrom}\ z\rangle$$
$$\left\{\begin{array}{l} x.\ z \hookrightarrow_n \mathsf{Some}\ \mathit{to} * \\ \quad (x = \texttt{None} \vee (\exists m.\, x = \texttt{Some}\, (\mathsf{body}(m), \mathsf{from}(m)) * \Phi(m) * \mathsf{R}(m))) \end{array}\right\}$$

When trying to receive a message on a socket, either a message will be received or no message is available. This is reflected directly in the logic: if no message was received, no resources are obtained. If a message $m$ is received, the resources prescribed by $\Phi(m)$ are transferred together with an unmodifiable certificate $\mathsf{R}(m)$ accounting logically for the fact that message $m$ was received. This certificate can in the logic be used to talk about messages that has actually been received in contrast to arbitrary messages. In our specification of the two-phase commit protocol presented in Sect. 6, the notion of a vote denotes not just a message with the right content but only one that has been sent by a participant and received by the coordinator.

### 4.2 Adequacy for Aneris

We now state a formal adequacy theorem, which expresses that Aneris guarantees both safety, and, that all protocols are adhered to.

To state our theorem we introduce a notion of *initial state coherence*: A set of addresses $A \subseteq \mathit{Address} = \mathit{Ip} \times \mathit{Port}$ and a map $\mathcal{P} : \mathit{Ip} \xrightarrow{\text{fin}} \wp^{\text{fin}}(\mathit{Port})$ are said to satisfy initial state coherence if the following hold: (1) if $(i, p) \in A$ then $i \in \text{dom}(\mathcal{P})$, and (2) if $i \in \text{dom}(\mathcal{P})$ then $\mathcal{P}(i) = \emptyset$.

**Theorem 1 (Adequacy).** *Let $\varphi$ be a first-order predicate over values, i.e., a meta logic predicate (as opposed to Iris predicates), let $\mathcal{P}$ be a map $\mathit{Ip} \xrightarrow{\mathit{fin}} \wp^{\text{fin}}(\mathit{Port})$, and $A \subseteq \mathit{Address}$ such that $A$ and $\mathcal{P}$ satisfy initial state coherence. Given a primordial socket protocol $\Phi_a$ for each $a \in A$, suppose that the Hoare triple*

$$\left\{\mathsf{Fixed}(A) * \mathop{\Huge *}_{a \in A} a \Mapsto \Phi_a * \mathop{\Huge *}_{i \in \text{dom}(\mathcal{P})} \mathsf{FreeIp}(i)\right\} \langle n_1; e\rangle \{v.\varphi(v)\}$$

*is derivable in Aneris.*

*If we have*

$$(\langle n_1; e\rangle, (\emptyset, \emptyset, \mathcal{P}, \emptyset)) \twoheadrightarrow^* ([\langle n_1; e_1\rangle, \langle n_2; e_2\rangle, \dots \langle n_m; e_m\rangle], \Sigma)$$

*then the following properties hold:*

1. *If $e_1$ is a value, then $\varphi(e_1)$ holds at the meta-level.*
2. *Each $e_i$ that is not a value can make a node-local, thread-local reduction step.*

Given predefined socket protocols for all primordial protocols and the necessary free IP addresses, this theorem provides the normal adequacy guarantees of Iris-like logics, namely *safety*, i.e., that nodes and threads on nodes cannot get stuck and that the postcondition holds for the resulting value. Notice, however, that this theorem also implies that all nodes adhere to the agreed upon protocols; otherwise, a node not adhering to a protocol would be able to cause another node to get stuck, which the adequacy theorem explicitly guarantees against.

## 5 Case Study 1: A Load Balancer

AnerisLang supports concurrent execution of threads on nodes through the fork $\{e\}$ primitive. We will illustrate the benefits of node-local concurrency by presenting an example of server-side load balancing.

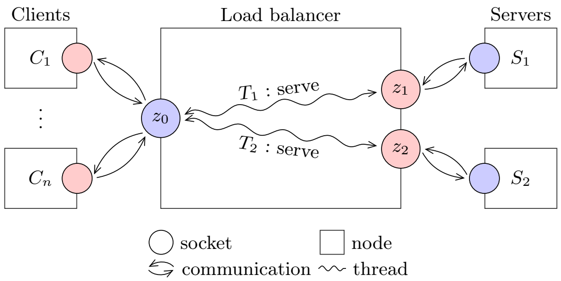

**Fig. 4.** The architecture of a distributed system with a load balancer and two servers.

*Implementation.* In the case of server-side load balancing, the work distribution is implemented by a program listening on a socket that clients send their requests to. The program forwards the requests to an available server, waits for the response from the server, and sends the answer back to the client. In order to handle requests from several clients simultaneously, the load balancer can employ concurrency by forking off a new thread for every available server in the system that is capable of handling such requests. Each of these threads will then listen for and forward requests. The architecture of such a system with two servers and $n$ clients is illustrated in Fig. 4.

An implementation of a load balancer is shown in Fig. 5. The load balancer is parameterized over an IP address, a port, and a list of servers. It creates a socket (corresponding to $z_0$ in Fig. 4), binds the address, and folds a function over the list of servers. This function forks off a new thread (corresponding to $T_1$ and $T_2$ in Fig. 4) for each server that runs the serve function with the newly-created socket, the given IP address, a fresh port number, and a server as arguments.

The serve function creates a new socket (corresponding to $z_1$ and $z_2$ in Fig. 4), binds the given address to the socket, and continuously tries to receive a client request on the main socket ($z_0$) given as input. If a request is received, it forwards the request to its server and waits for an answer. The answer is passed on to the client via the main socket. In this way, the entire load balancing process is transparent to the client, whose view will be the same as if it was communicating with just a single server handling all requests itself as the load balancer is simply relaying requests and responses.

*Specification and Protocols.* To provide a general, reusable specification of the load balancer, we will parameterize its socket protocol by two predicates $P_{in}$ and $P_{out}$ that are both predicates on a message $m$ and a meta-language value

```
rec load_balancer ip port servers =
  let skt = socket () in
  let a = makeaddress ip port in
  socketbind skt a;
  listfold (λ server, acc.
    fork { serve skt ip acc server };
    acc + 1) 1100 servers
```

```
rec serve main ip port srv =
  let skt = socket () in
  let a = makeaddress ip port in
  socketbind skt a;
  (rec loop () =
    match receivefrom main with
      SOME m =>
        sendto skt (π1 m) srv;
        let res = π1 (listenwait skt) in
        sendto main res (π2 m); loop ()
    | NONE => loop ()
    end) ()
```

**Fig. 5.** An implementation of a load balancer in AnerisLang. `listfold` and `listenwait` are convenient helper functions available in the appendix [20].

$v$. The two predicates are application specific and used to give logical accounts of the client requests and the server responses, respectively. Furthermore, we parameterize the protocol by a predicate $P_{val}$ on a meta-language value that will allows us to maintain ghost state between the request and response as will become evident in following.

In our specification, the sockets where the load balancer and the servers receive requests (the blue sockets in Fig. 4) will all be governed by the same socket protocol $\Phi_{rel}$ such that the load balancer may seamlessly relay requests and responses between the main socket and the servers, without invalidating any socket protocols. We define the generic relay socket protocol $\Phi_{rel}$ as follows:

$$\Phi_{rel}(P_{val}, P_{in}, P_{out})(m) \triangleq \exists \Psi, v.\, \mathsf{from}(m) \Rrightarrow \Psi * P_{in}(m, v) * P_{val}(v) * \\ (\forall m'.\, P_{val}(v) * P_{out}(m', v) \mathrel{-\!\!*} \Psi(m'))$$

When verifying a request, this protocol demands that the sender (corresponding to the red sockets in Fig. 4) is governed by some protocol $\Psi$, that the request fulfills the $P_{in}$ and $P_{val}$ predicates, and that $\Psi$ is satisfied given a response that maintains $P_{val}$ and satisfies $P_{out}$.

When verifying the load balancer receiving a request $m$ from a client, we obtain the resources $P_{in}(m, v)$ and $P_{val}(v)$ for some $v$ according to $\Phi_{rel}$. This suffices for passing the request along to a server. However, to forward the server's response to the client we must know that the server behaves faithfully and gave us the response to the right request value $v$. $\Phi_{rel}$ does not give us this immediately as the $v$ is existentially quantified. Hence we define a ghost resource $\mathsf{LB}(\pi, s, v)$ that provides fractional ownership for $\pi \in (0, 1]$, which satisfies $\mathsf{LB}(1, s, v) \dashv\vdash \mathsf{LB}(\frac{1}{2}, s, v) * \mathsf{LB}(\frac{1}{2}, s, v)$, and for which $v$ can only get updated if $\pi = 1$ and in particular $\mathsf{LB}(\pi, s, v) * \mathsf{LB}(\pi, s, v') \implies v = v'$ for any $\pi$. Using this resource, the server with address $s$ will have $P_{LB}(s)$ as its instantiation of $P_{val}$ where

$$P_{LB}(s)(v) \triangleq \mathsf{LB}(\tfrac{1}{2}, s, v).$$

When verifying the load balancer, we will update this resource to the request value $v$ when receiving a request (as we have the full fraction) and transfer

$\mathsf{LB}(\frac{1}{2}, s, v)$ to the server with address $s$ handling the request and, according to $\Phi_{rel}$, it will be required to send it back along with the result. Since the server logically only gets half ownership, the value cannot be changed. Together with the fact that $v$ is also an argument to $P_{in}$ and $P_{out}$, this ensures that the server fulfills $P_{out}$ for the same value as it received $P_{in}$ for. The socket protocol for the `serve` function's socket ($z_1$ and $z_2$ in Fig. 4) that communicates with a server with address $s$ can now be stated as follows.

$$\Phi_{serve}(s, P_{out})(m) \triangleq \exists v.\, \mathsf{LB}(\tfrac{1}{2}, s, v) * P_{out}(m, v)$$

Since all calls to the `serve` function need access to the main socket in order to receive requests, we will keep the socket resource required in an invariant $I_{LB}$ which is shared among all the threads:

$$I_{LB}(n, z, a) \triangleq \boxed{z \hookrightarrow_n \mathsf{Some}\ a}$$

The specification for the `serve` function becomes:

$$\begin{Bmatrix} I_{LB}(n, main, a_{main}) * \mathsf{Dynamic}((ip, p), A) * \mathsf{IsNode}(n) * \mathsf{LB}(1, s, v) * \\ a_{main} \Mapsto \Phi_{rel}(\lambda\_.\mathsf{True}, P_{in}, P_{out}) * s \Mapsto \Phi_{rel}(P_{LB}(s), P_{in}, P_{out}) \end{Bmatrix}$$
$$\langle n; \texttt{serve}\ main\ ip\ p\ s \rangle$$
$$\{\mathsf{False}\}$$

The specification requires the address $a_{main}$ of the socket $main$ to be governed by $\Phi_{rel}$ with a trivial instantiation of $P_{val}$ and the address $s$ of the server to be governed by $\Phi_{rel}$ with $P_{val}$ instantiated by $P_{LB}$. The specification moreover expects resources for a dynamic setup, the invariant that owns the resource needed to verify use of the $main$ socket, and a full instance of the $\mathsf{LB}(1, s, v)$ resource for some arbitrary $v$.

With this specification in place the complete specification of our load balancer is immediate (note that it is parameterized by $P_{in}$ and $P_{out}$):

$$\left\{ \begin{array}{l} \mathsf{Static}((ip, p), A, \phi_{rel}(\lambda\_.\mathsf{True}, P_{in}, P_{out})) * \mathsf{IsNode}(n) * \\ \left( \mathop{\Huge *}_{p' \in ports} \mathsf{Dynamic}((ip, p'), A) \right) * \\ \left( \mathop{\Huge *}_{s \in srvs} \exists v.\ \mathsf{LB}(1, s, v) * s \Mapsto \phi_{rel}(P_{LB}(s), P_{in}, P_{out}) \right) \end{array} \right\}$$
$$\langle n; \texttt{load\_balancer}\ ip\ p\ srvs \rangle$$
$$\{\mathsf{True}\}$$

where $ports = [1100, \cdots, 1100 + |srvs|]$. In addition to the protocol setup for each server as just described, for each port $p' \in ports$ which will become the endpoint for a corresponding server, we need the resources for a dynamic setup, and we need the resource for a static setup on the main input address $(ip, p)$.

In the accompanying Coq development we provide an implementation of the addition service from Sect. 2.3, both in the single server case and in a load balanced case. For this particular proof we let the meta-language value $v$ be a pair of integers corresponding to the expected arguments. In order to instantiate the load balancer specification we choose

$$P_{in}^{add}(m, (v_1, v_2)) \triangleq \mathsf{body}(m) = \mathit{serialize}(v_1, v_2)$$
$$P_{out}^{add}(m, (v_1, v_2)) \triangleq \mathsf{body}(m) = \mathit{serialize}(v_1 + v_2)$$

with *serialize* being the same serialization function from Sect. 2.3. We build and verify two distributed systems, (1) one consisting of two clients and an addition server and (2) one including two clients, a load balancer and three addition servers. We prove both of these systems safe and the proofs utilize the specifications we have given for the individual components. Notice that $\Phi_{rel}(\lambda\_.\mathsf{True}, P_{in}^{add}, P_{out}^{add})$ and $\Phi_{add}$ from Sect. 2.3 are the same. This is why we can use the same client specification in both system proofs. Hence, we have demonstrated Aneris' ability and support for horizontal composition of the same modules in different systems.

While the load balancer demonstrates the use of node-local concurrency, its implementation does not involve shared memory concurrency, i.e., synchronization among the node-local threads. The appendix [20] includes an example of a distributed system, where clients interact with a server that implements a bag. The server uses multiple threads to handle client requests concurrently and the threads use a *shared* bag data structure governed by a lock. This example demonstrates Aneris' ability to support both shared-memory concurrency and distributed networking.

## 6 Case Study 2: Two-Phase Commit

A typical problem in distributed systems is that of consensus and distributed commit; an operation should be performed by all participants in a system or none at all. The *two-phase commit* protocol (TPC) by Gray [6] is a classic solution to this problem. We study this protocol in Aneris as (1) it is widely used in the real-world, (2) it is a complex network protocol and thus serves as a decent benchmark for reasoning in Aneris, and (3) to show how an implementation can be given a specification that is usable for a client that abstractly relies on some consensus protocol.

The two-phase commit protocol consists of the following two phases, each involving two steps:

1. (a) The coordinator sends out a vote request to each participant.
   (b) A participant that receives a vote request replies with a vote for either commit or abort.
2. (a) The coordinator collects all votes and determines a result. If all participants voted commit, the coordinator sends a global commit to all. Otherwise, the coordinator sends a global abort to all.

(b) All participants that voted for a commit wait for the final verdict from the coordinator. If the participant receives a global commit it locally commits the transaction, otherwise the transaction is locally aborted. All participants must acknowledge.

Our implementation and specification details can be found in the appendix [20] and in the accompanying Coq development, but we will emphasize a few key points.

To provide general, reusable implementations and specifications of the coordinator and participants implementing TPC, we do not define how requests, votes, nor decisions look like. We leave it to a user of the module to provide decidable predicates matching the application specific needs and to define the logical, local pre- and postconditions, $P$ and $Q$, of participants for the operation in question.

Our specifications use fractional ghost resources to keep track of coordinator and participant state w.r.t. the coordinator and participant transition systems indicated in the protocol description above. Similar to our previous case study, we exploit partial ownership to limit when transitions can be made. When verifying a participant, we keep track of their state and the coordinator's state and require all participants' view of the coordinator state to be in agreement through an invariant.

In short, our specification of TPC

- ensures the participants and coordinator act according to the protocol, *i.e.*,
  - the coordinator decides based on all the participant votes,
  - participants act according to the global decision,
  - if the decision was to commit, we obtain the resources described by $Q$ for all participants,
  - if the decision was to abort, we still have the resources described by $P$ for all participants,
- does not require the coordinator to be primordial, so the coordinator could change from round to round.

### 6.1 A Replicated Log

In a distributed replicated logging system, a log is stored on several databases distributed across several nodes where the system ensures consistency among the logs through a consensus protocol. We have verified such a system implemented on top of the TPC coordinator and participant modules to showcase vertical composition of complex protocols in Aneris as illustrated in Fig. 6. The blue parts of the diagram constitute node-local instantiations of the TPC modules invoked by the nodes to handle the consensus process. As noted by Sergey et al. [35], clients of core consensus protocols have not received much focus from other major verification efforts [7, 30, 40].

Our specification of a replicated logging system draws on the generality of the TPC specification. In this case, we use fractional ghost state to keep track of two related pieces of information. The first keeps a logical account of the log $l$ already

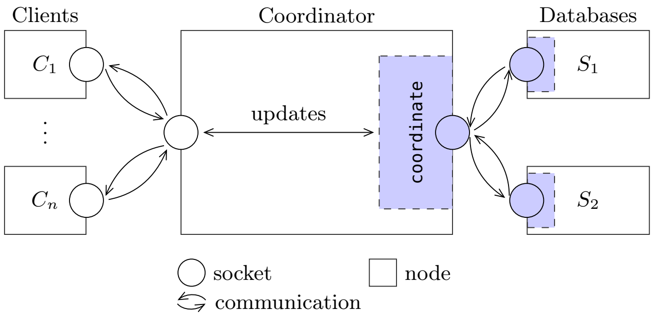

**Fig. 6.** The architecture of a replicated logging system implemented using the TPC modules (the blue parts of the diagram) with a coordinator and two databases ($S_1$ and $S_2$) each storing a copy of the log.

stored in the database at a node at address $a$, $\mathsf{LOG}(\pi, a, l)$. The second one keeps track of what the log should be updated to, if the pending round of consensus succeeds. This is a pair of the existing log $l$ and the (pending) change $s$ proposed in this round, $\mathsf{PEND}(\pi, a, (l, s))$. We exploit fractional resource ownership by letting the coordinator, logically, keep half of the pending log resources at all times. Together with suitable local pre- and postconditions for the databases, this prevents the databases from doing arbitrary changes to the log. Concretely, we instantiate $P$ and $Q$ of the TPC module as follows:

$$P_{rep}(p)(m) \triangleq \exists l, s.\, (m = \texttt{"REQUEST\_"} @ s) * \mathsf{LOG}(\tfrac{1}{2}, p, l) * \mathsf{PEND}(\tfrac{1}{2}, p, (l, s))$$
$$Q_{rep}(p)(n) \triangleq \exists l, s.\, \mathsf{LOG}(\tfrac{1}{2}, p, l@s) * \mathsf{PEND}(\tfrac{1}{2}, p, (l, s))$$

where @ denotes string concatenation. Note how the request message specifies the proposed change (since the string that we would like to add to the log is appended to the requests message) and how we ensure consistency by making sure the two ghost assertions hold for the same log. Even though $l$ and $s$ are existentially quantified, we know the logs cannot be inconsistent since the coordinator retains partial knowledge of the log. Due to the guarantees given by TPC specification, this implies that if the global decision was to commit a change this change will have happened locally on all databases, *cf.* $\mathsf{LOG}(\frac{1}{2}, p, l@s)$ in $Q_{rep}$, and if the decision was to abort, then the log remains unchanged on all databases, *cf.* $\mathsf{LOG}(\frac{1}{2}, p, l)$ in $P_{rep}$. We refer to the appendix [20] or the Coq development for further details.

## 7 Related Work

Verification of distributed systems has received a fair amount of attention. In order to give a better overview, we have divided related work into four categories.

*Model-Checking of Distributed Protocols.* Previous work on verification of distributed systems has mainly focused on verification of protocols or core network components through model-checking. Frameworks for showing safety and liveness properties, such as SPIN [9], and TLA+ [23], have had great success. A benefit of using model-checking frameworks is that they allow to state both safety and liveness assertions as LTL assertions [29]. Mace [17] provides a suite for building and model-checking distributed systems with asynchronous protocols, including liveness conditions. Chapar [25] allows for model-checking of programs that use causally consistent distributed key-value stores. Neither of these languages provide higher-order functions or thread-based concurrency.

*Session Types for Giving Types to Protocols.* Session types have been studied for a wide range of process calculi, in particular, typed $\pi$-calculus. The idea is to describe two-party communication protocols as a type to ensure communication safety and progress [10]. This has been extended to multi-party asynchronous channels [11], multi-role types [2] which informally model topics of actor-based message-passing and dependent session types allowing quantification over messages [38]. Our socket protocol definitions are quite similar to the multi-party asynchronous session types with progress encoded by having suitable ghost-assertions and using the magic wand. Actris [8] is a logic for session-type based reasoning about message-passing in actor-based languages.

*Hoare Style Reasoning About Distributed Systems.* Disel [35] is a Hoare Type Theory for distributed program verification in Coq with ideas from separation logic. It provides the novel protocol-tailored rules WithInv and Frame which allow for modularity of proofs under the condition of an inductive invariant and distributed systems composition. In Disel, programs can be extracted into runnable OCaml programs, which is on our agenda for future work.

IronFleet [7] allows for building provably correct distributed systems by combining TLA-style state-machine refinement with Hoare-logic verification in a layered approach, all embedded in Dafny [24]. IronFleet also allows for liveness assertions. For a comparison of Disel and IronFleet to Aneris from a modularity point of view we refer to the Introduction section.

*Other Distributed Verification Efforts.* Verdi [40] is a framework for writing and verifying implementations of distributed algorithms in Coq, providing a novel approach to network semantics and fault models. To achieve compositionality, the authors introduced *verified system transformers*, that is, a function that transforms one implementation to another implementation with different assumptions about its environment. This makes vertical composition difficult for clients of proven protocols and in comparison AnerisLang seems more expressive.

EventML [30, 31] is a functional language in the ML family that can be used for coding distributed protocols using high-level combinators from the Logic of Events, and verify them in the Nuprl interactive theorem prover. It is not quite clear how modular reasoning works, since one works within the model, however, the notion of a central main observer is akin to our distinguished system node.

## 8 Conclusion

Distributed systems are ubiquitous and hence it is essential to be able to verify them. In this paper we presented Aneris, a framework for writing and verifying distributed systems in Coq built on top of the Iris framework. From a programming point of view, the important aspect of AnerisLang is that it is feature-rich: it is a concurrent ML-like programming language with network primitives. This allows individual nodes to internally use higher-order heap and concurrency to write efficient programs.

The Aneris logic provides node-local reasoning through socket protocols. That is, we can reason about individual nodes in isolation as we reason about individual threads. We demonstrate the versatility of Aneris by studying interesting distributed systems both implemented and verified within Aneris. The adequacy theorem of Aneris implies that these programs are safe to run.

**Table 1.** Sizes of implementations, specifications, and proofs in lines of code. When proving adequacy, the system must be closed.

| Module | Implementation | Specification | Proofs |
|---|---|---|---|
| Load Balancer (Sect. 5) | | | |
| Load balancer | 18 | 78 | 95 |
| Addition Service | | | |
| Server | 11 | 15 | 38 |
| Client | 9 | 14 | 26 |
| Adequacy (1 server, 2 clients) | 5 | 12 | 62 |
| Adequacy w. Load Balancing (3 servers, 2 clients) | 16 | 28 | 175 |
| Two-phase commit (Sect. 6) | | | |
| Coordinator | 18 | 181 | 265 |
| Participant | 11 | | 280 |
| Replicated logging (Sect. 6 + appendix [20]) | | | |
| Instantiation of TPC | - | 85 | - |
| Logger | 22 | 19 | 95 |
| Database | 24 | 20 | 190 |
| Adequacy (2 dbs, 1 coordinator, 2 clients) | 13 | - | 137 |

Relating the verification sizes of the modules from Table 1 to other formal verification efforts in Coq indicates that it is easier to specify and verify systems in Aneris. The total work required to prove two-phase commit with replicated logging is 1,272 lines which is just half of the lines needed for proving the inductive invariant for TPC in other works [35]. However, extensive work has gone into Iris Proof Mode thus it is hard to conclude that Aneris requires less verification effort and does not just have richer tactics.

## Acknowledgments

This work was supported in part by the ModuRes Sapere Aude Advanced Grant from The Danish Council for Independent Research for the Natural Sciences (FNU); a Villum Investigator grant (no. 25804), Center for Basic Research in Program Verification (CPV), from the VILLUM Foundation; and the Flemish research fund (FWO).
